# Characterization of a novel chaperone/usher fimbrial operon present on KpGI-5, a methionine tRNA gene-associated genomic island in *Klebsiella pneumoniae*

**DOI:** 10.1186/1471-2180-12-59

**Published:** 2012-04-20

**Authors:** Jon J van Aartsen, Steen G Stahlhut, Ewan M Harrison, Marialuisa Crosatti, Hong-Yu Ou, Karen A Krogfelt, Carsten Struve, Kumar Rajakumar

**Affiliations:** 1 Department of Infection, Immunity and Inflammation, University of Leicester, Leicester, LE1 9HN, UK; 2 Department of Microbiological Surveillance and Research, Statens Serum Institut, Artillerivej 5, 2300, Copenhagen S, Denmark; 3 State Key Laboratory of Microbial Metabolism, Shanghai Jiaotong University, Shanghai, 200030, P.R. China; 4 Department of Clinical Microbiology, University Hospitals of Leicester NHS Trust, Leicester, LE1 5WW, UK

## Abstract

**Background:**

Several strain-specific *Klebsiella pneumoniae* virulence determinants have been described, though these have almost exclusively been linked with hypervirulent liver abscess-associated strains. Through PCR interrogation of integration hotspots, chromosome walking, island-tagging and fosmid-based marker rescue we captured and sequenced KpGI-5, a novel genomic island integrated into the *met56* tRNA gene of *K. pneumoniae* KR116, a bloodstream isolate from a patient with pneumonia and neutropenic sepsis.

**Results:**

The 14.0 kb KpGI-5 island exhibited a genome-anomalous G + C content, possessed near-perfect 46 bp direct repeats, encoded a γ_1_-chaperone/usher fimbrial cluster (*fim2*) and harboured seven other predicted genes of unknown function. Transcriptional analysis demonstrated expression of three *fim2* genes, and suggested that the *fim2A-fim2K* cluster comprised an operon. As fimbrial systems are frequently implicated in pathogenesis, we examined the role of *fim2* by analysing KR2107, a streptomycin-resistant derivative of KR116, and three isogenic mutants (Δ*fim*, Δ*fim2* and Δ*fim*Δ*fim2*) using biofilm assays, human cell adhesion assays and pair-wise competition-based murine models of intestinal colonization, lung infection and ascending urinary tract infection. Although no statistically significant role for *fim2* was demonstrable, liver and kidney CFU counts for lung and urinary tract infection models, respectively, hinted at an ordered gradation of virulence: KR2107 (most virulent), KR2107∆*fim2*, KR2107∆*fim* and KR2107∆*fim*∆*fim2* (least virulent). Thus, despite lack of statistical evidence there was a suggestion that *fim* and *fim2* contribute additively to virulence in these murine infection models. However, further studies would be necessary to substantiate this hypothesis.

**Conclusion:**

Although *fim2* was present in 13% of *Klebsiella* spp. strains investigated, no obvious *in vitro* or *in vivo* role for the locus was identified, although there were subtle hints of involvement in urovirulence and bacterial dissemination from the respiratory tract. Based on our findings and on parallels with other fimbrial systems, we propose that *fim2* has the potential to contribute beneficially to pathogenesis and/or environmental persistence of *Klebsiella* strains, at least under specific yet-to-be identified conditions.

## Background

*Klebsiella pneumoniae* is a Gram negative member of the Enterobacteriaceae family that commonly causes nosocomial pneumonia, bacteriaemia, urinary tract infections and wound infections [1]. In recent years the treatment of *K. pneumoniae* infections has become more challenging due to the greater prevalence of multiple antibiotic resistant strains [2,3]. Moreover, hypervirulent, pyogenic liver abscess-causing *K. pneumoniae* strains that infect otherwise healthy individuals have emerged from initial endemic foci in Taiwan and China, and are now spreading into North America and Europe [4-6]. This highlights the increasing threat that *K. pneumoniae* poses to public health and the importance of elucidating its mechanisms of pathogenesis.

Most *K. pneumoniae* strains possess a thick polysaccharide capsule which is involved in protection from opsonisation and phagocytosis and is a well recognized *in vivo* virulence factor [7]. Various studies have also highlighted roles for surface-exposed lipopolysaccharides, multiple iron acquisition systems and adhesins in *K. pneumoniae* infection [1,7,8]. Several strain-specific virulence determinants of the pyogenic liver abscess-associated isolate *K. pneumoniae* NTUH-K2044 have been well characterised [9-11]. However, the functions of strain-specific genomic regions in *K. pneumoniae* strains associated with other types of infection remain poorly studied.

Comparative analyses using computational and *in vitro* experimental techniques have shown that *K. pneumoniae* strains possess an extremely plastic genome that consists of a conserved core genome interspersed by strain-specific accessory components [12-15]. This was further highlighted in a recent study which calculated that only 54.7% of known *K. pneumoniae* genes were shared by three sequenced isolates (Kp342, MGH78578, NTUH-K2044) [15]. Genomic islands (GI), typically ranging from 10 kb to 200 kb in size and frequently inserted within tRNA gene (*tRNA*) hotspots, comprise a substantial proportion of the accessory genome. GI acquisition offers an efficient ‘quantum leap’ based route to gaining virulence factors, antibiotic resistance determinants and/or metabolic pathways pre-tailored for the exploitation of new environments [16,17].

Epidemiological studies have suggested that *K. pneumoniae* infections are preceded by colonization of the gastrointestinal tract [18]. Adhesion and colonization are essential steps in the infection process and are often mediated by fimbriae, which are small hair-like extensions on the bacterial cell surface that can interact with other surfaces via tip-located adhesin proteins [19]. The majority of environmental and clinical *K. pneumoniae* isolates are known to express type 1 fimbriae and type 3 fimbriae, which have recently been classified into the γ_1_ and γ_4_-fimbrial subgroups using the Nuccio and Bäumler fimbrial classification system, which was created from a large scale phylogenetic analysis of fimbrial usher proteins [20-23]. Recent *in vivo* experiments have demonstrated a role for *K. pneumoniae* type 1 fimbriae in urinary tract infections [22]. Although type 3 fimbriae have been shown to enhance biofilm formation and mediate attachment to bladder epithelium *in vitro*, the role of these structures *in vivo* has yet to be determined as an isogenic *mrk* knockout strain was as virulent as its wildtype parent in murine pneumonia and urinary tract infection models [23,24].

*K. pneumoniae* type 1 and type 3 fimbriae are both thought to assemble via the chaperone/usher (CU) assembly pathway which has been characterised in detail for the archetypal *E. coli* type 1 and P fimbriae [25]. Some CU fimbriae, such as the Kpc fimbriae of *K. pneumoniae* NTUH-K2044, are encoded by only a subset of strains and are thought to potentially correlate with tropism towards particular host tissues and infection types [26]. Many strain-specific fimbriae are encoded on tRNA gene-associated GIs, best illustrated by the *saftcfsefstd* and *stb* fimbrial operons of *Salmonella enterica* serovar Typhi strain CT18. This latter strain encodes an arsenal of twelve putative CU fimbrial operons that are hypothesized to correlate with adaptation to the human host [27]. The genomes of *K. pneumoniae* Kp342, MGH78578 and NTUH-K2044 contain nine, eleven and eight CU fimbrial operons, respectively, though the originally described type 1 and type 3 fimbrial operons are common to all three [26]. Apart from the serotype K1-associated *kpc* operon, no studies have investigated the *in vitro* and/or *in vivo* role of other *K. pneumoniae* accessory fimbrial operons. We now describe the identification, genetic characterization and initial functional analysis of a novel CU fimbrial operon (*fim2*) that is encoded on a previously unidentified GI, KpGI-5, found inserted within the *met56* tRNA gene of *K. pneumoniae* strain KR116.

## Results

### The KpGI-5 genomic island codes for a novel predicted chaperone/usher fimbrial system

Whilst screening five tRNA gene insertion hotspots in sixteen clinical *K. pneumoniae* isolates for strain-specific DNA using a technique called tRIP-PCR [13,14], we found that *K. pneumoniae* KR116 possessed an ‘occupied’ *met56* tRNA locus. tRIP-PCR using primers PR601 and PR647, which were designed to amplify across the *met56* tRNA locus, failed to amplify a product in KR116. Single genome-specific primer based walking from the conserved *met56* upstream flank yielded ~3 kb of novel sequence.

To capture and sequence this entire strain-specific island, we tagged the known *tRNA*-proximal arm of the island with a kanamycin resistance cassette using allelic exchange. A fosmid library of this tagged strain (KR116 ∆*fim2K*::kan) was then created and used to isolate kanamycin resistance cassette-bearing inserts by marker rescue. Two overlapping fosmids, pJFos-1 and pJFos-4, shown by end-sequencing to span the entire strain-specific region were sequenced to define this novel KR116 *met56*-specific GI that we designated KpGI-5.

**Figure 1:**
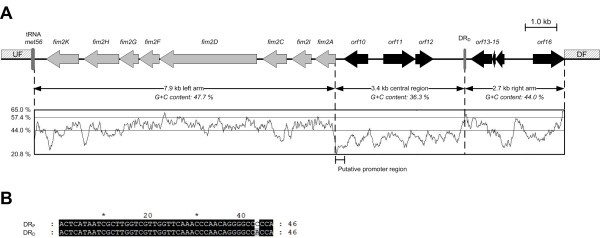
**Features of the novel KpGI-5 genomic island in*K. pneumoniae *KR116. (A)** Genetic organisation of KpGI-5 shown lying between the species-conserved upstream flank (UF) and downstream flank (DF) sequences. The eight putative fimbrial genes are labelled *fim2A*–*fim2K*. Closest BLASTP similarities for these and other predicted KpGI-5-encoded proteins are described in Table 1. KpGI-5 segments indicated by double arrows map to G + C % transitions as indicated by G + C profile. The thin horizontal lines on the G + C % graph represent the average G + C content of the *K. pneumoniae* MGH78578 genome (57.4%) and the entire KpGI-5 island (44.0%). The 20.8% and 65.0% G + C content lines correspond to the minimum and maximum G + C % calculated over an 80 bp window, respectively. **(B)** Alignment of the *tRNA*-proximal (DR_P_) and *tRNA*-distal (DR_D_) 46 bp direct repeat (DR) sequences associated with KpGI-5. DR_P_ comprises the 3’ end of *met56.*

KpGI-5 is a 14.0 kb insertion at the *met56* locus of KR116 with many features in common with typical GIs. Firstly, the calculated G + C content (44.0%) was much lower than the corresponding genome averaged values of *K. pneumoniae* MGH78578 (57.5%) and Kp342 (57.3%). Secondly, the island was present downstream of the *K. pneumoniae met56* gene, which is a proven hotspot for GI integration [15]. And finally, the island possessed an almost perfect 46 bp direct repeat that corresponded to the 3’ end of *met56*. However, no putative integrase or mobility-associated genes were identified. Open reading frame (ORF) and BLAST analyses were performed on the KpGI-5 sequence (Figure 1 and Table 1). The 2.7 kb segment mapping to the right arm of KpGI-5 was 90% identical to a region immediately downstream of *met56* in *K. pneumoniae* Kp342 and was predicted to encode two hypothetical proteins (Orf14 and Orf15), a metallo-beta-lactamase family protein (Orf16) and a putative GCN5-related N-acetyltransferase (Orf13). The nucleotide sequence of a 3.4 kb central region did not match any GenBank entries and coded for three novel proteins; Orf10 and Orf11 exhibited weak matches to putative regulatory proteins from the bacteria *Stigmatella aurantiaca* DW4/3-1 and *Serratia odorifera* DSM 4582, respectively. Orf10 also possessed a match to the pfam domain Trans_reg_C (PF00486) which has been implicated in DNA binding, further suggesting a role for Orf10 in regulation.

**Table 1:** **BLASTP homologs of proteins predicted to be encoded by KpGI-**5

**Gene name**	**Coding region (bp)**	**Protein size (aa**^**a**^**)**	**Percentage identity (aa**^**a**^**)**	**Organism**	**Possible function** **[GenBank Number]**	**E value**
*met56*	180..255 (76)	/	100% (note: BLASTN)	*K. pneumoniae* MGH78578	Methionine tRNA [KPN_03476]	/
*fim2K*	1385..528 (858)	285	60% (165/276)	*C. koseri* ATCC BAA-895	Putative EAL domain protein [ABV14791.1]	1e^-94^
*fim2H*	2440..1514 (927)	308	62% (190/308)	*K. pneumoniae* sp15	Fimbrial adhesin (FimH) [ACL13802.1]	1e^-101^
*fim2G*	2961..2458 (504)	167	72% (120/167)	*C. koseri* ATCC BAA-895	Minor fimbrial subunit (FimG) [ABV14789.1]	2e^-65^
*fim2F*	3501..2974 (528)	175	79% (138/175)	*C. koseri* ATCC BAA-895	Minor fimbrial subunit (FimF) [ABV14788.1]	1e^-73^
*fim2D*	6073..3515 (2559)	852	82% (689/838)	*C. koseri* ATCC BAA-895	Outer membrane usher protein (FimD) [ABV14787.1]	0.0
*fim2C*	6858..6229 (630)	209	92% (190/207)	*K. variicola* At-22	Fimbrial chaperone protein (FimC) [ADC56706.1]	2e^-107^
*fim2I*	7519..6989 (531)	176	82% (139/170)	*C. koseri* ATCC BAA-895	Fimbrial protein (FimI) [ABV14784.1]	2e^-80^
*fim2A*	8148..7600 (549)	182	88% (160/182)	*K. pneumoniae* MGH 78578	Major fimbrial protein (FimA) [ABR78685.1]	1e^-79^
*orf10*	9002..8355 (648)	215	37% (24/65)	*S. aurantiaca* DW4/3-1	Putative two component system regulatory protein [EAU69265.1]	0.019
*orf11*	9409..10254 (846)	281	28% (77/277)	*S. odorifera* DSM 4582	Putative transcriptional regulatory protein [EFE96725.1]	3e^-20^
*orf12*	10251..10727 (477)	158	29% (38/130)	*S. odorifera* DSM 4582	Hypothetical protein [EFE96270.1]	1e^-13^
*orf13*	12266..11694 (573)	190	97% (184/190)	*Klebsiella* sp. 1_1_55	Putative GCN5-related N-acetyltransferase [EFD84432.1]	1e^-106^
*orf14*	12387..12268 (120)	39	100% (39/39)	*K. pneumoniae* 342	Hypothetical protein [ACI07992.1]	1e^-12^
*orf15*	12616.. 12359 (234)	77	92% (71/77)	*K. pneumoniae* 342	Hypothetical protein [ACI06987.1]	1e^-34^
*orf16*	13342..14187 (846)	281	91% (256/281)	*K. pneumoniae* 342	Metallo-beta-lactamase family protein [ACI07748.1]	1e^-151^

^a^ aa, amino acids.

The 7.9 kb left arm of KpGI-5 harboured a novel eight-gene cluster that exhibited sequence similarity and organizational-identity to the chromosomally-encoded *fim* operons of *Citrobacter koseri* ATCC BAA-895 (~60%) and *K. pneumoniae* C3091 (~51%). This cluster was named *fim2*. It encoded homologs of all structural and biosynthesis-associated components of the well-characterized C3091 type 1 fimbrial system, including a major fimbrial subunit (Fim2A), three minor fimbrial subunits (Fim2F, Fim2G and Fim2H), and a chaperone (Fim2C) and usher (Fim2D) protein [22]. Downstream of *fim2H* was *fim2K* which encoded a FimK homolog that possessed a matching EAL domain but lacked a FimK-equivalent N-terminal helix-turn-helix domain. EAL domains have been implicated in the hydrolysis of c-di-GMP, an intracellular messenger that regulates important cellular functions including different forms of motility, adhesin and exopolysaccharide matrix synthesis, fimbrial expression and virulence [28-32]. Helix-turn-helix domains are associated with binding to specific DNA sequences and in the context of EAL domain-bearing proteins are hypothesized to modulate the c-di-GMP hydrolytic activity of these proteins [30]. Amino acid sequence identities between cognate *fim2* and *fim* products varied from 60 – 92%. However, no homologs of the C3091 *fimBfimE* or *fimS* invertible promoter switch could be identified upstream of *fim2*. *K. pneumoniae* KR116 also possessed the species-conserved *fim* and *mrk* operons, as shown by PCR screening for the *fimH* and *mrkD* adhesin genes using primer pairs PR1144-PR1145 and PR1150-PR1151, respectively. Of note, the G + C content of the *fim2* operon (47.7%) was much lower than that of the *K. pneumoniae fim* operon (60.8%) and quite distinct from the G + C content of the four fully sequenced *K. pneumoniae* genomes (56.9% – 57.4%).

### The KpGI-5 *fim2* locus is found within several *Klebsiella* spp. and is globally distributed

To determine the prevalence of *fim2* in *Klebsiella* spp., a total of 162 strains (123 *K. pneumoniae*, 19 undefined *Klebsiella* spp., 18 *K. oxytoca*, one *K. ornithinolytica* and one *K. planticola*) isolated from distinct sources were PCR screened for *fim2K* using primers PR615-PR616. In total, 21 out of 162 strains (13.0%) were identified to be *fim2* positive, including 16 *K. pneumoniae* (16/123 = 13.0%), three undefined *Klebsiella* spp. (3/19 = 15.7%) and two *K. oxytoca* (2/18 = 11.1%). It must be noted that these species designations are based on biochemical species identifications, which can be problematic in this genus [33]. 93.4% (15/16) of *fim2*-positive *K. pneumoniae* strains were also found to be *mrk-* and *fim-*positive by PCR analysis. However, the distribution of the latter were not investigated in other *Klebsiella* spp. due to recognized species-specific differences in *fim* and *mrk* operon sequences [34].

Further examination suggested that the specimen type from which strains were obtained was not a predictor of the presence or absence of *fim2* (Table 2). Notably, *fim2*-positive strains were not limited to one geographical area. KR116, the index *fim2*-positive strain, was isolated in the United Kingdom, while other *fim2*-bearing strains were isolated in Germany, Denmark, USA and China, suggesting a sporadic but global spread of the *fim2* locus.

**Table 2:** **Prevalence of *fim2 *by specimen type**

	**Total**^**a**^	***fim2*+**^**b**^	**Percentage^c^**
Ascitic fluid	9	1	11.1%
Biliary fluid	1	0	0%
Blood	48	8	16.7%
Cerebrospinal fluid	2	0	0%
Environmental	11	1	9.0%
Pyogenic liver abscess aspirates	11	0	0%
Nasopharynx	3	0	0%
Sputum	11	1	9.0%
Unknown	20	4	20.0%
Urine	45	5	11.1%
Wound	1	1	100%
All	162	21	13.0%

^a^ Total number of strains tested.

^b^ Total number of strains testing *fim2*-positive using primers PR615 and PR616.

^c^ Percentage of *fim2*-positive strains.

### *Fim2* genes are expressed under standard *in vitro* growth conditions

**Figure 2:**
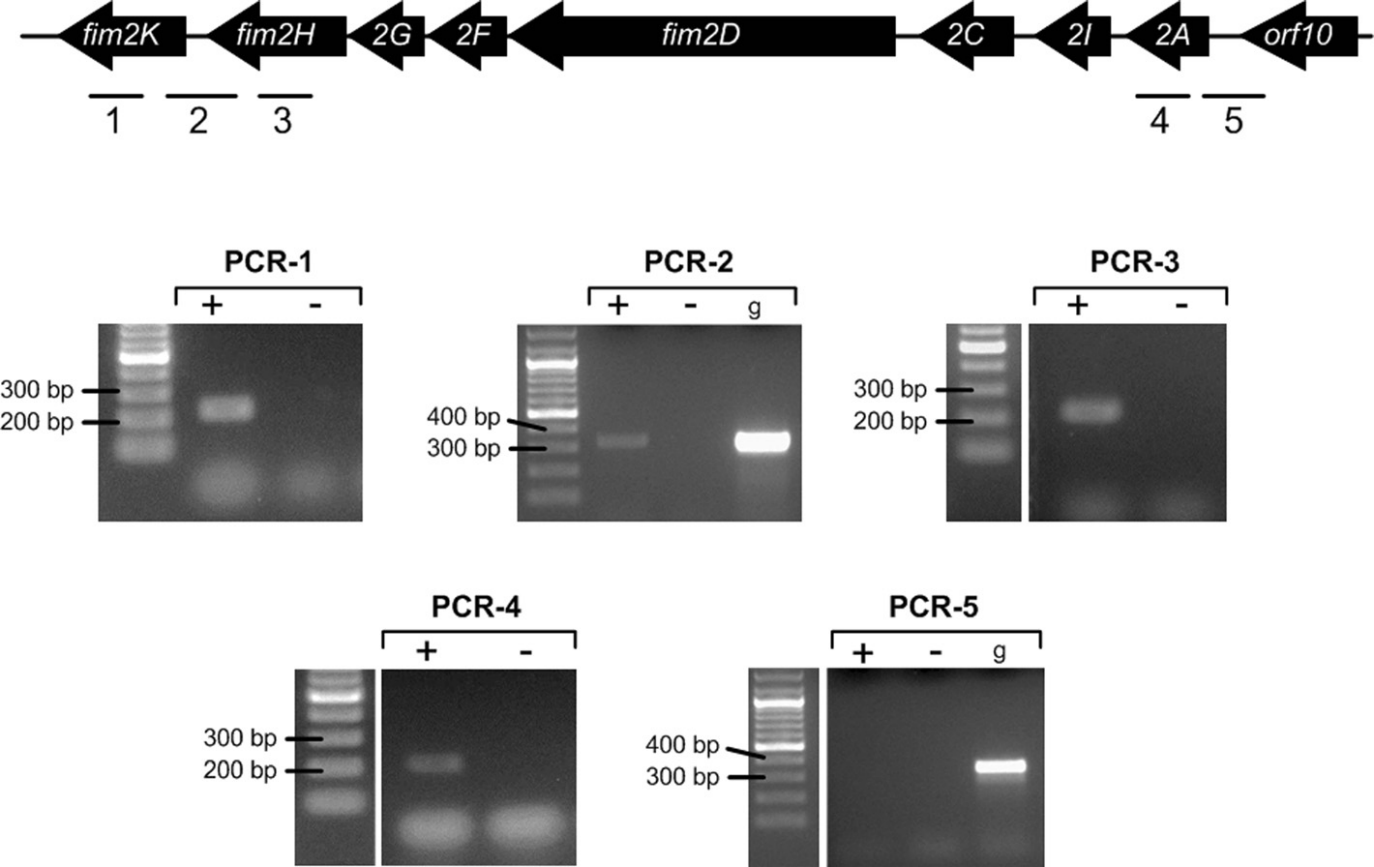
**Transcriptional analysis of *fim2*.** A schematic map of the *fim2* cluster and the upstream *orf10* gene to show regions targeted for transcriptional analysis: *fim2K* (PCR-1, 220 bp: PR1611/PR1612), *fim2H-fim2K* (PCR-2, 316 bp: PR16268/PR1629), *fim2H* (PCR-3, 241 bp: PR1609/PR1610), *fim2A* (PCR-4, 221 bp: PR1607/PR1608) and *fim2A-orf10* (PCR-5, 380 bp: PR1626/PR1627). RNA purified from an *in vitro* grown culture of KR2107 (LB, 37°C, 200 rpm, 16 h) was processed in parallel with (+) or without (−) reverse transcriptase and analysed by PCR with the primers listed above. KR2107 genomic DNA (g) and PCR-grade water (Neg) were used as PCR controls when necessary. Amplicons were visualised on 1.5% agarose gels. Distinct PCR amplicons were obtained for four of the five assays. The PCR-5 assay which sought to define a shared *orf10* and *fim2A* transcript was negative.

Many chaperone/usher operons are poorly expressed under laboratory conditions [35,36]. To investigate *fim2* expression, RNA was isolated from KR2107, a streptomycin-resistant derivative of KR116, which had been cultured in LB medium for 16 h (37°C, 200 rpm) and a cDNA library constructed using random primer-based RT-PCR. Subsequent PCR analysis of this cDNA library detected transcripts that corresponded to *fim2Afim2H* and *fim2K*, while reverse transcriptase-free control reaction mixtures did not yield any products, thus confirming absence of DNA carryover (Figure 2). Follow-up quantitative-PCR experiments on this KR2107 cDNA library showed that under the growth conditions examined *fim2A* was expressed approximately 30- and 90-fold less than *fimA* and *mrkA*, respectively (data not shown). As PCR analysis spanning *orf10* to *fim2A* did not yield a product, whilst that linking *fim2H* to *fim2K* produced a specific band, it would appear that the eight gene *fim2* cluster was expressed as a single transcript and that *orf1*0 gene was not part of this transcriptional unit (Figure 2). Of note, transcripts corresponding to *fim2Afim2H* and *fim2K* were also detected in KR2107 grown at 37°C for 16 h (200 rpm) in M9 minimal media plus 0.2% glycerol, RPMI 1640, RPMI 1640 plus 10% fetal calf serum and King’s B medium (data not shown).

### Heterologous expression of *fim2* does not result in visualisable host fimbriation

**Figure 3:**
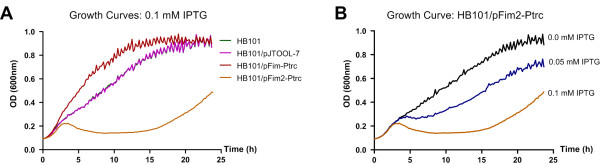
**IPTG induction of HB101/pFim2-Ptrc causes a major growth reduction. (A)** Growth curves for HB101, HB101/pJTOOL-7 (empty vector), HB101/pFim-Ptrc and HB101/pFim2-Ptrc. The growth curves for HB101 and HB101/pJTOOL-7 are largely superimposed as these are very similar. **(B)** Growth curves for HB101/pFim2-Ptrc grown for 24 h in LB broth containing 100 μg/ml ampicillin supplemented with 0.0 mM, 0.05 mM or 0.1 mM IPTG. Data shown in all cases represent the means of two biological replicates, each assayed in seven wells (n = 14).

The *fim2* locus was PCR-amplified from KR116 and cloned into the high copy number vector pBluescript II KS+, the low copy number vector pWSK129 and the P_TRC_-bearing vector pJTOOL-7 to create pFim2-HCN, pFim2-LCN and pFim2-Ptrc, respectively. Each plasmid was transformed into the afimbriate *E. coli* strain HB101 and examined by electron microscopy in an attempt to visualise the putative Fim2 fimbriae. Despite use of multiple induction methods and over 100 cells being viewed per strain, no definite fimbrial structures could be identified on the bacterial surfaces examined. Similar results were obtained when the locus was expressed in a *fim2*-negative *K. pneumoniae* mutant, C3091Δ*fim*Δ*mrk*. By contrast, HB101 possessing a pJTOOL-7 derivative with the *fim* operon expressed abundant and highly characteristic type 1 fimbriae on its outer surface. Notably, despite the absence of detectable fimbriation in *E. coli* HB101/pFim2-Ptrc induced with IPTG, major induction-associated growth reduction was observed (Figure 3A). HB101/pFim2-Ptrc growth inhibition exhibited a distinct dose–response relationship to IPTG concentration and this was not evident with the control strains HB101 and HB101/pJTOOL-7 (Figure 3B). By contrast, over-expression of *fim* appeared to enhance the growth rate of HB101/pFim-Ptrc but had no effect on final cell densities as compared to the above mentioned control strains.

### Expression of *fim2* in *E. coli* HB101 appears to enhance biofilm formation

**Figure 4:**
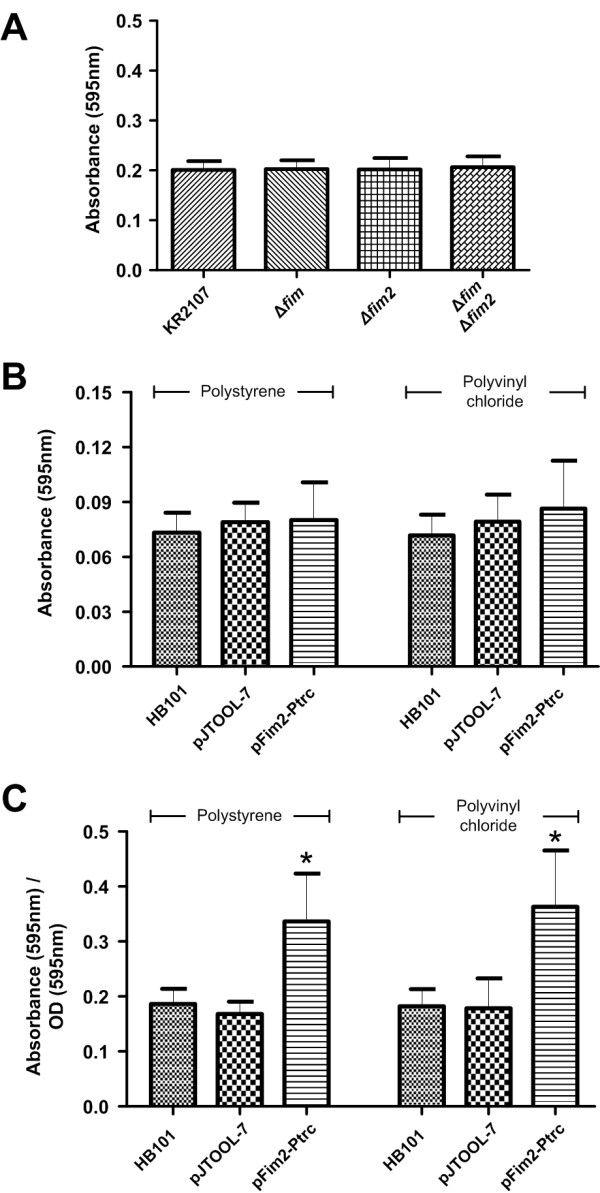
**The *fim2 *locus appears to contribute to biofilm formation when expressed in *E. coli *HB101. (A)** Results for biofilm formation assay on polystyrene for KR2107 and its three *fim* and/or *fim2* isogenic mutants as determined by crystal violet absorbance data. Equivalent results, suggestive of no strain-to-strain differences, were obtained for assays on polyvinyl chloride plates (data not shown). **(B)** Biofilm formation assay based on heterologous expression of *fim2* in *E. coli* HB101/pFim2-Ptrc. HB101 and HB101 carrying an empty pJTOOL-7 served as controls. Biofilm formation was quantified using crystal violet staining and absorbance was measured at 595 nm. Non-normalized crystal violet absorbance data are shown. **(C)** Biofilm formation assay results shown in (B) were normalized to take account of pre-wash total cell numbers based on OD_595_ readings performed at 48 h, just prior to washing off non-surface adherent cells and crystal violet staining. Data shown in all cases represent means and standard deviations of three biological replicates, each assayed in eight wells (n = 24). An asterisk indicates a highly significant difference (*P* < 0.0001) from HB101 and HB101/pJTOOL-7.

*K. pneumoniae* readily colonizes and forms biofilms on abiotic surfaces such as urinary catheters and tracheal tubes [21,37]. As surface-expressed structures play a key role in biofilm formation, the ability of KR2107 and its isogenic mutants to form biofilms was examined. However, absence of *fim2* and/or *fim* had no effect on biofilm formation as assayed at 24 h under static growth conditions in LB or M9 media at either 37°C or 30°C (Figure 4A; data not shown). To detect a potential contribution to biofilm formation that may have been masked by low-level *fim2* expression or capsule-related physical hindrance of fimbrial function [38], *fim2* was over-expressed from pFim2-Ptrc in *E. coli* HB101 using 0.05 mM IPTG induction. Compared to HB101 carrying the empty pJTOOL-7 vector, HB101/pFim2-Ptrc exhibited similar biofilm formation at 48 h on polystyrene wells as assessed by post-washing crystal violet staining (Figure 4B). On the other hand, expression of *fim2* in HB101 resulted in marginally denser biofilm in polyvinyl chloride wells as compared to the vector-only control, but this was not statistically significant (*P* = 0.464; Figure 4B).

As HB101/pFim2-Ptrc grew to a much lower OD_595_ at 48 h than the other two strains, we also analysed the biofilm data as a ratio of crystal violet staining intensity to the pre-wash OD_595_ measurement that reflected total growth. This analysis suggested that the proportion of HB101/pFim2-Ptrc cells comprising biofilm growth as opposed to total growth (biofilm and planktonic cells) was almost twice that of HB101 and the vector only control strain (Figure 4C). Indeed, based on this ratio, *fim2* expression in HB101 exerted a highly significant positive impact on biofilm formation on both surfaces (*P* < 0.0001 in each case). By contrast when *fim2* was expressed in the Mrk- and Fim-deficient strain C3091∆*fim*∆*mrk* using this same system, no statistically significant accentuation in biofilm formation on either surface was observed (data not shown).

### Deletion of *fim2* does not affect adhesion to human HCT-8 ileocaecal or 5637 bladder epithelial cells

**Figure 5:**
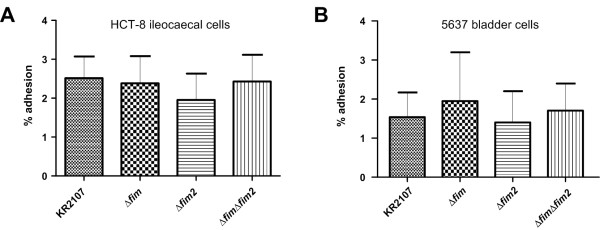
**Cell-adherence properties of *K. pneumoniae *KR2107 and its isogenic *fim*and/or *fim2 *mutants**. (A) *In vitro* adhesion assays to human HCT-8 ileocaecal cells. (B) *In vitro* adhesion assays to human 5637 bladder epithelial cells. In both cases percentages of bacteria that remained adherent to cell monolayers after 3 h of incubation at 37°C followed by careful washing are shown. Bars represent means and standard deviations.

*In vitro* adhesion assays were performed to further investigate whether KR2107 and its three isogenic mutants (KR2107∆*fim*, KR2107∆*fim2* and KR2107∆*fim*∆*fim2*) exhibited differing cell adhesion properties. Human HCT-8 ileocecal and human 5637 bladder epithelial cell lines were chosen to investigate adherence to intestine- and bladder-derived cells, respectively. No significant differences were detectable by these *in vitro* tissue culture assays (Figure 5). Furthermore, despite the previously reported impaired urovirulence of a *fim*-negative *K. pneumoniae* strain [22], the KR2107∆*fim* and KR2107∆*fim*∆*fim2* mutants examined in this study did not display any defect in adherence to bladder epithelial cells relative to KR2107 or KR2107∆*fim2*. It is possible that *fim* and/or *fim2* expression was insignificant under the *in vitro* conditions used or that the *K. pneumoniae* capsule interfered with fimbrial function [38,39].

### Deletion of *fim2* does not affect murine intestinal colonization

**Figure 6:**
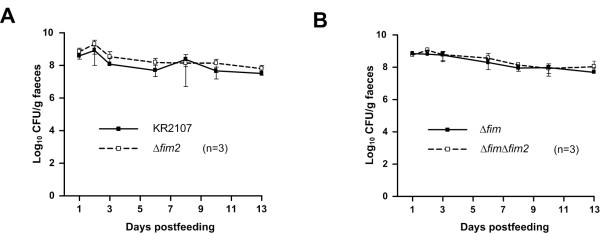
**Murine intestinal colonization of*K. pneumoniae *KR2107 and its isogenic *fim *and/or *fim2 *mutants. (A)** Intestinal co-colonization following oral feeding with a 1:1 mixture of KR2107 and KR2107∆*fim2*. **(B)** Intestinal co-colonization following oral feeding with a 1:1 mixture of KR2107∆*fim* and KR2107∆*fim*∆*fim2*. Mean CFU/g faeces and corresponding standard deviation values are shown.

Epidemiological studies have elucidated that the first step in the majority of *K. pneumoniae* infections is gastrointestinal tract colonization [18]. To investigate whether *fim2* influences this initial step, a 1:1 mixture of KR2107 and KR2107∆*fim2* was fed to three mice and faecal CFU counts were monitored for 13 days. To exclude potential type 1 fimbriae-related masking, a competition experiment between KR2107∆*fim* and KR2107∆*fim*∆*fim2* was also performed. As assessed by faecal CFU counts, no strain exhibited an obvious competitive advantage and all four strains were found to readily colonize the large intestine in similar numbers (~10^8^ – 10^9^ CFU/g) throughout the experiment (Figure 6). Apart from confirming that *fim* does not affect intestinal colonization [22], these results also suggested that *fim2* does not play a significant role in murine intestinal colonization by *K. pneumoniae*.

### The *fim2* locus is not a virulence factor in a murine lung infection model

**Figure 7:**
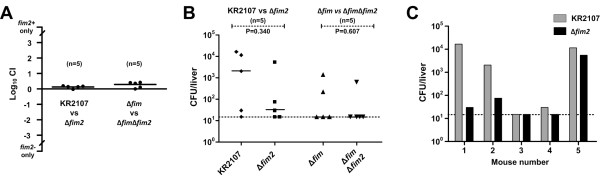
**Murine lung infection model studies with KR2107 and its isogenic *fim *and/or *fim2 *mutants. (A)** Comparison of the ability of KR2107 and its isogenic mutants to infect the lungs as assessed by two head-to-head competition assays. A mixture containing an equal ratio of each competing strain was inoculated intranasally into five mice. The competitive index (CI) is the ratio of the number of *fim2*-positive to *fim2*-negative bacteria recovered from infected organs divided by the equivalent ratio as present in the intranasal inoculum. **(B)** Differential CFU counts for each of the competing strains in the liver at 30 h post-inoculation. **(C)** Liver CFU counts obtained in the five mice used for the competition assay between KR2107 and its isogenic *fim2* mutant. In A and B, horizontal bars represent the median, with data points for each mouse as indicated. The lower limit of detection is represented by the dotted line. *P* values were calculated using the Mann–Whitney *U* test.

*K. pneumoniae* is a clinically important cause of lung infections and various potential virulence factors have been determined [40,41]. The influence of *fim2* on pneumovirulence was investigated by intranasal inoculation of five mice with a mixture comprising equal numbers of KR2107 and KR2107∆*fim2.* An equivalent competition experiment between KR2107∆*fim* and KR2107∆*fim*∆*fim2* was also performed*.* 30 h post-infection all mice displayed significant signs of disease and were sacrificed. High numbers of *K. pneumoniae* were found in the lungs of all mice (5 × 10^5^ – 1 × 10^7^ CFU/lung). Similar lung CFU counts were obtained for both competition assays. Furthermore, no significant deviation in *fim2*-positive to *fim2*-negative strain ratios was evident for either competition assay (Figure 7A). These data suggest that both *fim* and *fim2* do not impact significantly on pneumovirulence of *K. pneumoniae* in a murine lung infection model.

Total liver and spleen CFU counts were used as a measure of the ability of bacteria to disseminate from the lungs into the bloodstream. Much lower numbers and greater mouse-to-mouse variation occurred in CFU counts for the livers (<15 – 1.6 × 10^4^) and spleens (<20 – 200) of these mice. The median CFU count per liver for KR2107 (2.1 × 10^3^) was elevated compared to that of KR2107∆*fim2* (3.0 × 10^1^), although this difference was not significant (*P* = 0.340). When liver CFU counts were examined individually for each mouse, two mice exhibited greater than 1-log more KR2107 than KR2107∆*fim2*, while the difference, though still hinting at an advantage for KR2107, was less than 0.5 log for two other mice (Figure 7B and C). The liver CFU counts in mouse 3 for both strains were equal to the lower limit of detection and extrapolated from a single colony each, thus preventing meaningful comparison of these values. No difference was found between the median CFU counts per liver for KR2107∆*fim* and KR2107∆*fim*∆*fim2* (1.5 × 10^1^). Thus, despite the absence of firm conclusions emanating from these data, the possibility that *fim2* may play a role in systemic dissemination and/or survival of *K. pneumoniae* following murine lung infection cannot be dismissed entirely.

### Role of *fim2* in a murine urinary tract infection model

**Figure 8:**
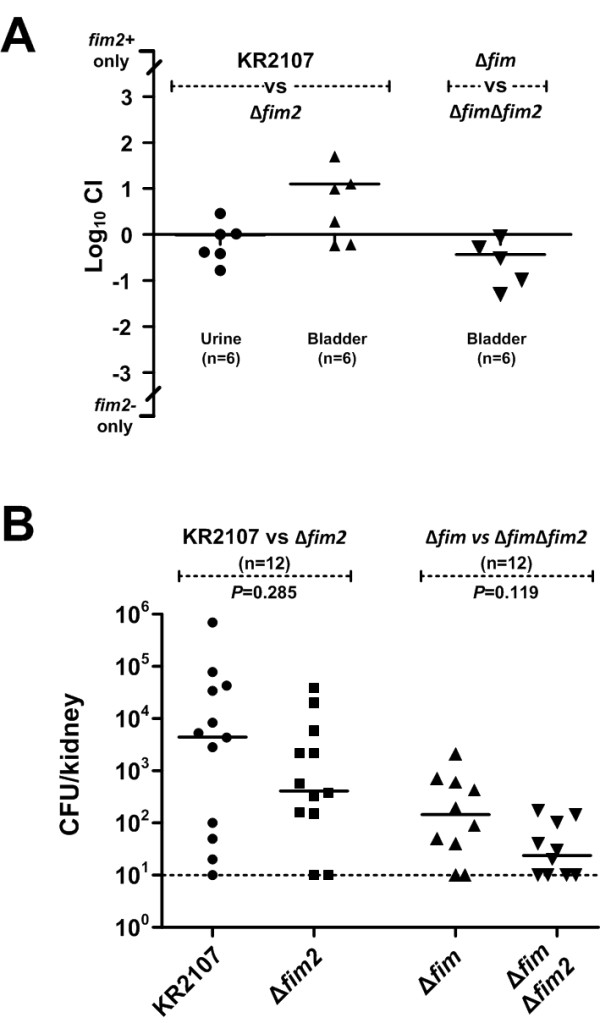
**Murine urinary tract infection model studies with KR2107 and its isogenic*fim*and/or*fim2*mutants. (A)** Comparison of the urovirulence of KR2107 and its isogenic mutants as assessed by two head-to-head competition assays. A mixture containing approximately equal numbers of each competing strain was inoculated into the bladders of six mice. The competitive index (CI) is the ratio of the number of *fim2*-positive to *fim2*-negative bacteria recovered from urine or bladder divided by the equivalent ratio as present in the infecting inoculum. **(B)** Differential CFU counts for each of the competing strains in the left and right kidneys at 3 days post-inoculation. In both of the above analyses horizontal bars represent the median, with data points for each mouse as indicated. The lower limit of detection is represented by the dotted line. *P* values were calculated using the Mann–Whitney *U* test.

Type 1 fimbriae are a well-established virulence factor of *K. pneumoniae* urinary tract infections [22,23]. To assess the role of *fim2* in *K. pneumoniae* urinary tract infection, a group of six mice were inoculated transurethrally with a 1:1 mixture of KR2107 and its *fim2* mutant and sacrificed 3 days post-inoculation. All urine and bladder samples were found to be colonized and a median CFU count of 8.7 × 10^5^ per bladder and 5.0 × 10^4^ per ml of urine was obtained. In all mice the infection had ascended into the kidneys producing a median bacterial count of 5.3 × 10^3^ per kidney (n = 12). The median CI value obtained for bladder samples indicates 10-fold more CFUs of KR2107 than the *fim2* mutant (Figure 8A). These values are supported by the median kidney CFU count which was 10-fold higher for the wildtype (4.8 × 10^3^) than the *fim2* mutant (4.8 × 10^2^), although this difference is not statistically significant (*P* = 0.285) (Figure 8B). Nevertheless, these concordant findings would suggest that *fim2* may exert a subtle influence on the urovirulence of *K. pneumoniae*.

To investigate potential genetic redundancy or functional masking between *fim* and *fim2*, the competition assay was repeated in a *fim*-negative background. Consistent with previous data [23], bacterial counts were considerably lower in this *fim*-negative background experiment as compared to the initial competition assay. Infection was established in the bladders of five out of six mice, with a median bacterial count of 1.35 × 10^2^ in these five mice. At time of sacrifice, infection had ascended into nine of ten kidneys with a median CFU count of 2.7 × 10^2^ (n = 10). However, in all cases no bacteria were isolated from the urine suggesting counts of less than 50 per ml. The median CI value obtained for bladder samples showed that CFU counts for KR2107∆*fim* and KR2107∆*fim*∆*fim2* did not differ significantly (Figure 8A). However, the median kidney CFU counts were 5.6-fold higher for the KR2107∆*fim* (1.4 × 10^2^) than KR2107∆*fim*∆*fim2* mutant (2.5 × 10^1^), and although similar to the results obtained in the *fim*-positive background these results were also not statistically significant (*P* = 0.066) (Figure 8B). These results have confirmed the importance of *fim* in *K. pneumoniae*-mediated urovirulence and further support the case for a potential but subtle accessory role for *fim2* in this disease process.

## Discussion

The plastic nature of *K. pneumoniae* genomes is well described and an increasing number of studies have elucidated the function of various components of the accessory genome of the pyogenic liver abscess-associated strain *K. pneumoniae* NTUH-K2044. However, functional characterization of the accessory genome of strains associated with other types of infection is lacking. In order to investigate the plasticity of *K. pneumoniae* associated with other infections, we previously interrogated the *pheV* locus of sixteen clinical isolates from patients without pyogenic liver abscesses for the presence of foreign DNA elements [13]. In this study, further tRIP-PCR interrogation of *K. pneumoniae* KR116 using *met56*-specific primers identified a novel GI, KpGI-5, inserted within its *met56* gene. KR116 had been isolated from the blood of a patient with pneumonia and neutropenic septicaemia. KpGI-5 was sequenced in this study and found to encode a putative γ_1_-type CU fimbrial operon that has been named *fim2*.

The genetic organization of *fim2* resembles that of the *K. pneumoniae fim* operon and contains homologs of all eight *fim* genes. *fim2* is predicted to code for a major fimbrial subunit (Fim2A), three minor fimbrial subunits (Fim2F, Fim2G, Fim2H) and homologs of the FimC and FimD chaperone and usher proteins, respectively, thus classifying this locus as a novel γ_1_-type CU operon that putatively encodes a fimbrial appendage [20]. A seventh predicted protein, Fim2I, exhibited 82% identity to FimI, a protein required for fimbrial biogenesis; however, the exact nature of this dependence remains unknown [42]. Amino acid sequences of the eight *fim2* gene products showed 60 to 92% identity to cognate Fim proteins. Indeed, the two clusters would appear to be pseudoparalogs, homologs that appear to be paralogous but have ended up in the same genome by both vertical and horizontal gene transfer [43]. The unique evolutionary origins of the *fim* and *fim2* cluster are further highlighted by differences in transcriptional control. The *fim* cluster is largely controlled by the FimB and FimE recombinases which together switch transcription on and off by inverting a 314 bp promoter-containing sequence called *fimS* that lies upstream of *fimA*[22]. Exact copies, genetic remnants or potential functional-replacements of the 9 bp *fimS*-flanking inverted repeats could not be identified within the putative *fim2* promoter region that lies upstream of *fim2A*. Furthermore, as KpGI-5 lacks homologs of the FimB and FimE recombinases we conclude that *fim2* expression is not controlled via a *fimS*-like switch mechanism. Additionally, the *fim2K* gene within the *fim2* cluster encodes an EAL domain-containing protein that is similar to FimK, which has previously been shown to regulate type 1 fimbrial expression [31]. FimK was hypothesised to exert its influence via the hydrolysis of the intracellular messenger c-di-GMP, which is known to regulate expression of virulence genes, motility and biofilm formation in other bacteria [29]. The *in vitro* and *in vivo* function of Fim2K is currently under investigation.

Bacterial adhesion to and colonization of host cells is frequently mediated by a diverse assortment of afimbrial and fimbrial adhesins, each thought to possess a particular tissue tropism [19]. The vast majority of *K. pneumoniae* strains are able to produce type 1 fimbriae [37,44]. These structures are associated with mannose-sensitive agglutination of guinea pig red blood cells, a phenotype caused by interaction of the adhesin subunit FimH with terminally-exposed mannose residues in N-linked oligosaccharides on cell surfaces [45]. Previously it has been shown that the FimH residues partaking in binding to mono- and tri-mannose moieties are highly conserved [45]. The specific binding properties of Fim2H, the putative Fim2 adhesin, remain to be identified but it is unlikely to bind to mannose since only four out of the 13 mono- and tri-mannose binding residues of FimH are strictly conserved in Fim2H [45]. This is also in agreement with the inability of *E. coli* HB101 expressing *fim2* to agglutinate guinea pig red blood cells (data not shown), though the relevance of these data remain uncertain given the lack of visualisable fimbriae in this model.

Despite multiple attempts we were unable to visualize fimbrial structures using electron microscopy when the *fim2* operon was over-expressed in *E. coli* HB101 and *K. pneumoniae* C3091Δ*fim*Δ*mrk*. Paradoxically, biofilm forming ability appeared to be enhanced in this *fim2*-expressing *E. coli* strain. These results are similar to those of a study in which constitutive expression of four of seven *E. coli* CU fimbrial operons was shown to cause phenotypic alternations despite the fact that fimbrial appendages could not be visualized by electron microscopy [36]. Difficulty in visualisation of fimbriae by electron microscopy has also been described for the enterotoxigenic *E. coli* fimbriae CS3 and CS6 and the putative Stg fimbriae of *Salmonella enterica* serovar Typhi [46-48]. Most interestingly, when the latter was expressed in a bald *E. coli* strain an enhanced ability to adhere to INT-407 epithelial cells was noted despite the absence of EM-observable fimbriae [48]. It is possible that the *fim2* operon may code for a short and/or thin fimbrial structure that is not readily visualized by electron microscopy, or one that is extremely fragile. Conceivably, the hypothesized Fim2 appendages may be best expressed under biofilm-forming conditions, potentially explaining the enhanced biofilm-forming phenotype exhibited by HB101/pFim2-Ptrc, or in other specific *in vivo* environments. Alternatively, the putative phosphodiesterase Fim2K may regulate *fim2* transcription and/or that of an unknown *E. coli* adherence factor via a c-di-GMP-dependent pathway. Indeed, heterologous expression of *fim2K* has been shown to complement a mutant lacking an EAL-bearing protein (van Aartsen and Rajakumar, unpublished data). Proposed future anti-Fim2A-based immunofluorescence and immunogold electron microscopy studies in addition to detailed characterisation of Fim2K will ultimately help determine the mechanism by which *fim2* contributes to biofilm formation.

The genomes of *E. coli* K-12, *E. coli* O157:H7 and *Salmonella* Typhi possess numerous cryptic CU fimbrial operons that are tightly regulated and not expressed under the majority of *in vitro* conditions tested [35,36,49]. In this work, *fim2*-specific transcript was identified in standard laboratory culture but the amount detected was 30- to 90-fold lower than that identified for *fim* and *mrk*, respectively. Compared to the *K. pneumoniae* genome-averaged A + T content (~43%), *fim2* is AT-rich (53%) and the putative promoter region upstream of *fim2A* possesses an even higher AT-content (73%). As moderate-to-marked upregulation of seven CU fimbrial operons has been reported in an *E. coli* K-12 H-NS mutant [36], the finding of an AT-rich *fim2* promoter region suggests that the H-NS protein may play a role in controlling this operon as well. Moreover, H-NS has been shown to bind preferentially to regions of horizontally-acquired DNA in *Salmonella* Typhimurium and it is therefore possible this also occurs with KpGI-5 [50]. Furthermore, in addition to Fim2K, KpGI-5 also encodes two other potential regulators one or more of which could alter *fim2* expression. By analogy with other CU systems, we propose that upregulation of *fim2* expression and biosynthesis of Fim2 fimbriae is likely to be triggered by specific environmental conditions and involve a complex interplay of multiple transcriptional regulators such as H-NS, Fim2K and/or FimK, and levels of expression of other surface components, such as the capsule [31,36,38,51]. It is important to note that even though *fim2* lacks an invertible promoter switch, it may still be stochastically controlled by a bistable regulatory circuit such as the DNA methylation-based system described in detail for *E. coli* Pap fimbriae and it is therefore possible that single cell variants expressing *fim2* may exist [51].

Analysis of three sequenced *K. pneumoniae* strains revealed that, in addition to the *fim* and *mrk* operons, these genomes collectively encode at least six other CU fimbrial systems [22,23], one or more of which may perform an as-yet uncharacterised role in adhesion to target tissues. To investigate the role of *fim2* in virulence, isogenic *fim2* mutants were constructed and examined in three murine models, each focussed on primary infection of a distinct clinically-relevant anatomical site. Surprisingly, despite many fimbrial systems having been clearly implicated in virulence, we detected no clear evidence of attenuation (murine lung and urinary tract infection models) or reduction in colonizing ability (murine intestinal colonization model) in the *fim2*-negative strains studied.

Intriguingly, examination of bladder CFU count-based CIs for the urinary tract infection experiments hinted at a subtle role for *fim2* in the colonization of bladder and kidney tissues. In both tissues, median wildtype CFU counts were approximately ten-fold higher than those of the *fim2* mutant, although when performed in a *fim* negative background this difference was reversed and reduced in bladder and kidney samples, respectively. Nevertheless, the latter conflicting results may due to the markedly lower CFU counts obtained in the *fim* negative background. As shown by neutral CI values in the lung tissue but an approximately 100-fold higher median liver CFU count for KR2107 as compared to its isogenic *fim2* mutant, the *fim2* locus would appear to be involved in systemic dissemination and/or survival of *K. pneumoniae* following primary infection of the respiratory tract. However, given the noted lack of statistical significance, low numbers of mice examined and substantial mouse-to-mouse variation for these liver CFU data, no firm conclusions can be derived at present. As an aside, the previously demonstrated dramatic positive contribution of *fim* to urovirulence in this murine model was also shown to be the case in the KR2107 background [22,23]. At an overview level, based on total CFU counts per liver and per kidney for the lung infection and ascending urinary tract infection models, respectively, there was a suggestion, though not supported statistically, of an ordered gradation amongst the four isogenic strains with the most-to-least virulent as follows: KR2107, KR2107∆*fim2*, KR2107∆*fim* and KR2107∆*fim*∆*fim2*. We speculate this relates to a Fim2-mediated enhancement of bacterial biofilm-forming-, adhesive- and/or invasive-potential under the *in vivo* conditions tested. In addition, the predicted influence of Fim2K on the c-di-GMP regulatory circuit, may itself impact on virulence via regulation of Fim2, Fim and/or other virulence factors.

The *fim2* cluster was also assessed for its ability to contribute to biofilm formation. Gene knock-out experiments in KR2107 failed to reveal a role for *fim2* in biofilm formation. However, the function of the product of *fim2* may have been masked due to physical interference by the *K. pneumoniae* capsule, a phenomenon previously observed with type 1 fimbriae [38,39]. Alternatively, it may be a function of limited *fim2* expression under the *in vitro* conditions examined. Therefore, heterologous expression of *fim2* in the afimbriate *E. coli* strain HB101 and the bald *fim2*-negative *K. pneumoniae* C3091∆*fim*∆*mrk* mutant was pursued*.* Yet again evidence of a *fim2*-associated phenotype was elusive and only apparent in HB101 and then too only when crystal violet-staining data were standardised for total pre-wash cell numbers. Attempts to alleviate the observed growth retardation associated with over-expression of *fim2* in a HB101 background by reducing incubation temperature to 30°C and by providing rare tRNAs *in trans* were unsuccessful. Furthermore, the observed growth retardation was highly reproducible even when newly generated HB101 strains possessing independently-constructed pFim2-Ptrc plasmids were used instead (van Aartsen and Rajakumar, unpublished data). Thus, it would appear that over-expression of *fim2* in HB101 was specifically responsible for this phenotype, though no comparable effect occurred with over-expression of *fim*.

The presence of *fim2* in more than one species and its global spread suggests that this horizontally acquired locus has been maintained within a subset of the *Klebsiella* population due to positive selection. Hence, although the role *fim2* remains elusive, given the glimpses of functionality hinted at by our data and the evolutionary survival of this multi-gene entity, we hypothesize that putative Fim2 contributes to pathogenesis of infection and/or environmental persistence, at least under highly specific conditions.

## Conclusions

In conclusion, we have described the KpGI-5 island which possessed a novel γ_1_-type CU operon called *fim2*. Although *fim2* was shown to be expressed at an mRNA level and its function was investigated using three distinct murine infection models, tissue culture experiments and biofilm assays, no obvious *in vitro* or *in vivo* role for the *fim2* locus was identified, although there were subtle hints of involvement in urovirulence and in bacterial dissemination from the respiratory tract. Nevertheless, as *fim2* was found in approximately 13% of *Klebsiella* spp. isolates examined, we propose that *fim2* has the potential to contribute beneficially to its host *Klebsiella* strains at least under specific conditions.

## Methods

### Bacterial strains, plasmids, and growth media

Bacterial strains and plasmids used in this study are described in Table 3. *K. pneumoniae* KR116 is a human blood stream infection isolate obtained from the University Hospitals of Leicester. Unless otherwise specified, strains were routinely cultured at 37°C in LB medium supplemented with 50 μg/ml apramycin, 250 μg/ml ampicillin, 30 μg/ml chloramphenicol, 50 μg/ml kanamycin and/or 15 μg/ml tetracycline for *K. pneumoniae*, and 100 μg/ml ampicillin, 12.5 μg/ml chloramphenicol, 50 μg/ml kanamycin and/or 10 μg/ml tetracycline for *E. coli*.

**Table 3:** Bacterial strains and plasmids used in this study

**Bacterial strain or plasmid**	**Relevant characteristics**^**a**^	**Reference or Source**
**Bacterial strains**		
*Escherichia coli*		
DH5α	F- φ80d*lacZ*∆M15 ∆(*lacZYA-argF*)U169 *deoR recA1endA1 hsdR17*(rK-mK+) *phoA supE*44 λ- *thi*-1 *gyrA96 relA1*	[52]
HB101	F- *mcrB mrrhsdS20*(rB- mB-) *recA13 leuB6ara*-14 *proA2 lacY1galK2 xyl*-5 *mtl*-1 *rpsL20*(Sm^R^) glnV44 λ-	[53]
CC118λ*pir*	Δ(*are-leu*) *araD* Δ*lacX74 galEgalK phoA20thi*-1 *rpsE rpoBargE*(Am) *recA1* λ*pir*	[54]
S17-1λ*pir*	F’ *recA hsdR* RP4-2 (Tc::Mu) (Km::Tn7) λ*pir*	[55]
EPI300-T1R	F- *mcrA* Δ(*mrr*-*hsdRMS*-*mcrBC*) φ80d*lacZ*ΔM15 Δ*lacX74 recA1endA1 araD139* Δ(*ara-leu*)7697 *galU galK* λ- *rpsL nupGtrfA tonAdhfr*	Epicentre
*Klebsiella pneumoniae*		
C3091	Clinical urinary tract infection isolate; St^R^	[56]
C3091∆*fim*::tet∆*mrk*::kan	C3091 with *fim* and *mrk* clusters deleted; St^R^, Tet^R^, Kan^R^	[23]
KR116	Clinical blood stream infection isolate	This work
KR116 ∆*fim2K*::kan	KR116 with an insertion-deletion mutation in *fim2K* where 125 bp has been replaced by a kanamycin resistance cassette; Kan^R^	This work
KR2107	Spontaneous streptomycin resistant derivative of KR116; St^R^	This work
KR2107∆*fim*	KR2107 with *fim* deleted; St^R^, Tet^R^	This work
KR2107∆*fim2*	KR2107 with *fim2* deleted; St^R^, Kan^R^	This work
KR2107∆*fim*∆*fim2*	KR2107 with *fim* and *fim2* deleted; St^R^, Tet^R^, Kan^R^	This work
**Plasmids**		
pDS132	Lambda *pir*-based suicide vector; Cml^R^	[57]
pBluescript II KS+	High copy number cloning vector; Amp^R^	Fermentas
pWSK129	Low copy number cloning vector; Kan^R^	[58]
pKOBEG-Apra	Lambda Red expression plasmid, P_BAD_ promoter; Apr^R^	[59]
pRT733	Source of Kan^R^ cassette; Kan^R^	[60]
pCC2FOS	Fosmid cloning vector; Cml^R^	Epicentre
pTRC99a	IPTG inducible expression vector with P_TRC_ promoter and *lacI*^*q*^; Amp^R^	[61]
pJTOOL-7	Derivate of pTRC99a with an added NotI cut site in multiple the cloning site; Amp^R^	This work
pJKO-4a	pDS132 with SOE-PCR fragment for tagging of *fim2K* (*fim2K*::Kan); Cml^R^, Kan^R^	This work
pJFos-1	40.6 kb *fim2K*::Kan-containing fragment cloned into pCC2FOS; Cml^R^, Kan^R^	This work
pJFos-4	26.1 kb *fim2K*::Kan-containing fragment cloned into pCC2FOS; Cml^R^, Kan^R^	This work
pFim2-HCN	9.4 kb PCR fragment containing the *fim2* locus cloned into the NotI site of pBluescript II KS+; Amp^R^	This work
pFim2-LCN	9.4 kb PCR fragment containing the *fim2* locus cloned into the NotI site of pWSK129; Amp^R^	This work
pFim2-Ptrc	9.0 kb PCR fragment containing the *fim2* locus cloned into the NotI/SbfI site of pJTOOL-7. IPTG inducible; Amp^R^	This work

^a^**Amp ampicillin; Apr apramycin; Cml chloramphenicol; Kan kanamycin; St streptomycin; Tet tetracycline. Superscript R indicates resistance.**

### DNA analysis and manipulations

Restriction enzymes and T4 DNA ligase were obtained from New England Biolabs and/or Promega and used according to manufacturer’s instructions. Genomic DNA and plasmid DNA were extracted using the ArchivePure DNA Purification Kit (5 PRIME) and GenElute™ Plasmid Miniprep Kit (Sigma), respectively. Primers used in this study are listed in Additional file 2: Table S1. Standard PCR amplification was carried out using GoTaq (Promega).

An 8.2 kb region containing the *fim2* operon (*fim2A*-*fim2K*) was amplified from KR116 with primers PR937 and PR938 using KOD Hot Start polymerase (Merck), and cloned into the NotI site of pBluescript II KS + and pWSK129 to create high (pFim2-HCN) and low copy number (pFim2-LCN) plasmid clones, respectively. Additionally, the *fim2* locus was amplified using primers PR1224 and PR1222 and was cloned into pJTOOL-7, a pTRC99a derivative, to create pFim2-Ptrc. A fosmid library representative of KR116 ∆*fim2K*::kan was constructed using the Epicentre Copy Control Fosmid Library Production kit, with some minor modifications. Briefly, 2.5 μg of genomic DNA was sheared to ~40 kb fragments by pipetting through a 200 μl tip. After end repair, the DNA was ligated into pCC2FOS and packaged into phages using MaxPlax Lambda Packaging Extracts (Epicentre) which were then used to infect *E. coli* EPI300-T1R. Marker rescue of kanamycin resistant fosmid clones was performed by plating infected EPI300-T1R cells on LB plates supplemented with chloramphenicol and kanamycin. Selected fosmids were subjected to approximately 60-fold coverage Roche 454 pyrosequencing (University of Leicester NUCLEUS Genomics Core Facility).

### Construction of mutant strains

*K. pneumoniae* KR2107, a spontaneous streptomycin-resistant mutant of KR116, was used as the parent strain for all isogenic mutants. It possessed a 24 h growth curve identical to KR116 and agglutinated guinea pig red blood cells in a similar manner. *fim2* was exchanged for a kanamycin resistance cassette by lambda Red-mediated recombination. KR2107 was transformed with pKOBEG-Apra, a temperature-sensitive plasmid encoding the lambda Red recombinase system, and grown at 30°C in LB media supplemented with apramycin and 0.2% arabinose. Electrocompetent KR2107/pKOBEG-Apra cells were prepared according to standard methods and electroporated with an SOE-PCR product comprising a kanamycin resistance gene cassette and targeting flanking homologous sequences (Additional file 1: Figure S1). The KR2107∆*fim2* mutant was obtained by selecting on LB media plus kanamycin at 37°C. Loss of pKOBEG-Apra was confirmed by reversion to apramycin sensitivity and a negative PCR with primers EBGNHe and EBGh3. The KR2107∆*fim2* mutant was validated by PCR analysis using primer pairs PR1103-Kn2 (2590 bp) and Kn1-PR1104 (3903 bp). The 2095 bp ∆*fim*::tet fragment was amplified from C3091∆*fim*::tet∆*mrk*::kan using primers UpfimB-F and DwfimK-R and electroporated into arabinose-induced KR2107/pKOBEG-Apra to construct the *fim* mutant [23]. KR2107∆*fim*∆*fim2* was constructed similarly from a KR2107∆*fim*/pKOBEG-Apra intermediate strain. KR116 ∆*fim2K*::kan was constructed by conjugative transfer of the suicide construct pJKO-4a to facilitate allelic exchange ([62]; Additional file 1: Figure S1).

### Transcriptional analysis of *fim2*

Total RNA was prepared from KR2107 after growing for 16 h in LB liquid medium (37°C, 200 rpm) using the Norgen Total RNA Purification Kit. The Ambion TURBO DNA-free kit was used to remove residual DNA and cDNA libraries synthesised using the QuantiTect Reverse Transcription kit (Qiagen) as recommended by the manufacturer. An identical reaction without reverse transcriptase was performed to assess DNA contamination. Regions corresponding to *fim2A*, *fim2H* and *fim2K* were PCR amplified using primers pairs PR1607-PR1608, PR1609-PR1610, and PR1611-PR1612, respectively. Regions linking *116met56-10* to *fim2A* and *fim2H* to *fim2K* were detected using primer pairs PR1626-PR1627 and PR16268-PR1629, respectively. Amplicons were visualised on 1.5% agarose gels.

### Transmission electron microscopy

Five μl of sample was applied to a hydrophilic Formvar-carbon coated copper grid (Agar Scientific) and allowed to adsorb for 5 min. After wicking excess liquid, the grid was washed once using distilled deionised water and then negative-stained for 15 s with a droplet of 1% uranyl acetate (pH 4.5). Electron microscopy was performed on a JEOL JEM-1400 microscope at 80 kV.

### Biofilm, growth curve and epithelial adhesion assays

Biofilm assays were performed using a modified microtiter plate-based method [63]. Briefly, strains were grown for 16 h (37°C, 200 rpm) in LB broth with antibiotics if necessary and subcultured 1:100 into 100 μl LB medium with 0.05 mM IPTG and ampicillin, when required, in 96-well microtiter plates (Nunc). Plates were incubated statically for 48 h at 37°C and OD_595_ (optical density at 595 nm) readings obtained at the end of incubation. Following incubation the medium was removed and the plate washed once with distilled water. 125 μl of 0.1% (v/v) crystal violet was added to each well and left to stain for 10 min. The plate was then washed twice with distilled water, dried thoroughly and the stain eluted with 200 μl of 95% ethanol per well and the absorbance measured at 595 nm (BioRad Model 680 Microplate reader). Each was strain tested in eight wells and three replicate experiments were performed.

Growth curves were performed similarly to biofilm assays with a few minor modifications. Plates were incubated statically for 24 h at 37°C in a Varioskan (Thermo Scientific) instrument. The plates were subjected to a brief vigorous shake every 10 min immediately prior to the absorbance being measured at 600 nm (OD_600_). Each strain was tested in seven wells and two duplicate experiments were performed.

Quantitative assessment of bacterial adhesion to epithelial cells was performed using human HCT-8 ileocaecal and 5637 bladder cells. HCT-8 cells were subcultivated (1:10) twice a week in RPMI 1640 medium containing 25 mM HEPES, 2 mM glutamine, 1 mM pyruvate, 10% fetal calf serum, 0.002% neomycin and 0.01% streptomycin. 5637 cells were cultivated similarly but no pyruvate was added to the medium. Epithelial cells were seeded into two 24-well tissue culture plates (Nunc) and grown to confluent monolayers. After carefully washing each well three times with warm PBS, 1 ml of fresh supplement-free RPMI 1640 was added and inoculated with ~2 × 10^6^ CFU from an overnight culture. Plates were incubated for 3 h at 37°C. One plate was then used to determine the total number of bacteria at the end of 3 h incubation, as described previously [56]. The wells in the second plate were carefully washed three times with PBS and then used to determine the total number of adherent bacteria. All assays were performed in duplicate and repeated independently four times.

### Murine models of infection

Six- to eight-week-old female CFW1 mice (Harlan) were used for intestinal colonization experiments as described previously [64]. Briefly, mice were provided with drinking water containing 5 g/l streptomycin sulphate for 24 h and fed a 100 μl suspension containing ~10^9^ CFU of each strain in 20% sucrose. On indicated days, faecal pellets were collected, weighed and homogenised in 0.9% NaCl and dilutions plated onto MacConkey agar supplemented with appropriate antibiotics for faecal CFU counts.

A previously described intranasal infection model was used in a co-infection format [23]. Six- to eight-week-old female NMRi mice (Harlan) were anaesthetized and hooked on a string by their front teeth. 50 μl of bacterial suspension containing ~5 × 10^7^ CFU of each strain was dropped onto the nares to allow for aspiration. Mice were left hooked on the string for 10 min before being returned to their cages. At sacrifice lungs, spleen and liver were collected in 0.9% NaCl and homogenised. Serial dilutions were plated on selective media for CFU counts.

The ascending urinary tract infection model in which C3H mice (Harlan) were inoculated transurethrally with 50 μl of bacterial suspension containing ~5 × 10^8^ CFU bacteria has been described in detail previously [22,65]. All animal experiments were conducted under the auspices of the Animal Experiments Inspectorate, the Danish Ministry of Justice.

### Data analysis, statistics and nucleotide accession number

Nucleotide sequences were annotated and analysed using the Integrative Services for Genomic Analysis software and manually curated [66]. The competitive index (CI) was calculated by dividing the ratio of *fim2*-positive to *fim2*-negative bacteria recovered from infected organs by the ratio of the corresponding bacteria in the initial inoculum. The non-parametric Mann–Whitney *U* test was used to analyse infection data. Biofilm and cell-adhesion data were analysed using the non-parametric Kruskal-Wallis test and Dunn’s posthoc analysis. The nucleotide sequence of KpGI-5 has been deposited online [GenBank: JN181158].

## Authors’ contributions

JJvA carried out the molecular genetic studies, *in vitro* assays and bioinformatics analyses. CAS and JJvA carried out the murine infection studies. MC performed the growth curve experiments. EMH and HYO participated in experimental design and bioinformatics analyses. KR and JJvA conceived the study, participated in its design and coordination and drafted the manuscript. SGS, KAK and CS contributed to experimental design and analysis. All authors read, contributed to and approved the final manuscript.

## Supplementary Material

Additional file 1 **Figure S1.** Details of SOE-PCR products used for targeted mutagenesis in this study. Click here for file

Additional file 2 **Figure S1.** Study Oligonucleotide primers used in this study. Click here for file
