# Supplementary material for: Characterization of a novel chaperone/usher fimbrial operon present on KpGI-5, a methionine tRNA gene-associated genomic island in Klebsiella pneumoniae
Source: BMC Microbiol. 2012 Apr 20;12:59. doi: 10.1186/1471-2180-12-59 (PMC3419637; doi:10.1186/1471-2180-12-59)

# Figure S1: Details of SOE-PCR products used for targeted mutagenesis in this study.

## A) *fim2*::kan – used for lambda Red recombination

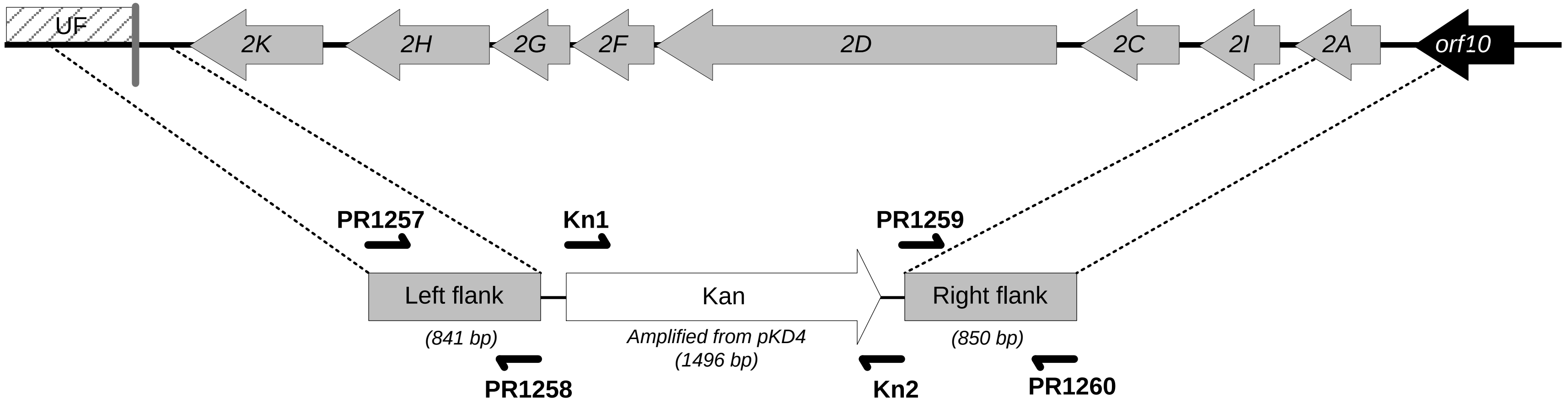


## B) *fim2K*::kan – cloned into the XbaI restriction site of pDS132 to make suicide vector pJKO-4a

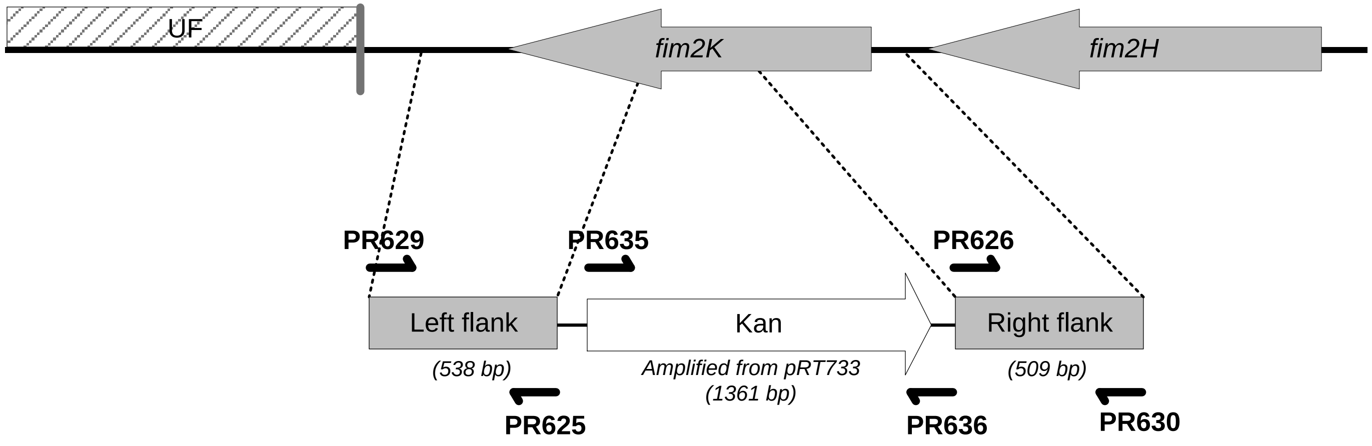

Supplement: Additional file 1 — Figure S1. Details of SOE-PCR products used for targeted mutagenesis in this study. [file 1471-2180-12-59-S1.pdf]
