# Supplementary material for: Characterization of a novel chaperone/usher fimbrial operon present on KpGI-5, a methionine tRNA gene-associated genomic island in Klebsiella pneumoniae
Source: BMC Microbiol. 2012 Apr 20;12:59. doi: 10.1186/1471-2180-12-59 (PMC3419637; doi:10.1186/1471-2180-12-59)
Supplement: Additional file 2 — Figure S1. Study Oligonucleotide primers used in this study. [file 1471-2180-12-59-S2.pdf]

**Table S1 – Oligonucleotide primers used in this study**

| Name | Sequence (5' to 3') |
|---|---|
| UpfimB-F | TGCGGGTATCATCAAGAG |
| DwfimK-R | CGATAACACCCGCGAATACGAC |
| EBGh3 | GGGAAGCTTATTATCGTGAGGATGCGTCA |
| EBGNHe | CCCGCTAGCGAAAAGATGTTTCGTGAAGC |
| Kn1 | GTGTAGGCTGGAGCTGCTTC |
| Kn2 | ATGGGAATTAGCCATGGTCC |
| PR601 | GGTTGATACCGCAAAAGACAA |
| PR615 | TCAACCGGCTGCGAAAACAAAT |
| PR616 | CAAGAAGCGGTGCAGGGTATCAA |
| PR625 | TCCGGTTCGCATGCATTCCCCAGCGTGATTTTTCCGATAAG |
| PR626 | TCTCATGAGTATGCATTCCCGGCCCACAGCGGATTATG |
| PR629 | GCTCTAGAGTAATGGGATGGCGACAGG |
| PR630 | GCTCTAGAGGCGTCGTCCAAACTCTAC |
| PR635 | GGGAATGCATGCGAACCGGAATTGC |
| PR636 | GGGAATGCATACTCATGAGATGCC |
| PR647 | GGCGAGGATATTATTCATTCCC |
| PR937 | GCGCGGCCGCGCAATTCTCTGACATTGATGCT |
| PR938 | GCGCGGCCGCACAACTTAGCTATCAAACTCGG |
| PR1103 | TATCGACATGAACACCGACC |
| PR1104 | GAACCGACTATATTCATTCGAG |
| PR1144 | CACGCAAGGCACCATTC |
| PR1145 | GCTCAGAATCAACATCGGTAAC |
| PR1150 | AATAGCAGCCACGCGATAGT |
| PR1151 | TGTTTATCAGCGATGCGAAC |
| PR1222 | GGCGCGGCCGCTAATGCAAAAATTTTTGTAATTGC |
| PR1224 | CCACCTGCAGGCAATTCTCTGACATTGATGCT |
| PR1257 | CATCGTCCAGTTCACGATAGG |
| PR1258 | GAAGCAGCTCCAGCCTACACAACGGGATGTTTACAAAGCA |
| PR1259 | GGACCATGGCTAATTCCCATGAAAGCAACAGACGCTTTGG |
| PR1260 | GAAAGTTGTATGTTGGCATTGG |
| PR1607 | CGTTAATGGCGGTACAGTGC |
| PR1608 | CTGAGGTACCGGAGAAAGCA |
| PR1609 | CAGCGAGTAGTGTCGCGATT |
| PR1610 | TGTGCGCAATGAACGGTAAC |
| PR1611 | GCCATTCAGAACGGTCAGGT |

| | |
|---|---|
| PR1612 | ATTCGCCCTGATAGCGATTG |
| PR1626 | TTATACCTTAAATAAGCCAGTTGA |
| PR1627 | GTGGTATTAGCCAAAGCCGC |
| PR1628 | TCTTTGGGGACGGTAGGC |
| PR1629 | TGGCATTCTTTTCGGCTATC |
